# Exploring the Human Microbiome: The Potential Future Role of Next-Generation Sequencing in Disease Diagnosis and Treatment

**DOI:** 10.3389/fimmu.2018.02868

**Published:** 2019-01-07

**Authors:** Muneer Ahmad Malla, Anamika Dubey, Ashwani Kumar, Shweta Yadav, Abeer Hashem, Elsayed Fathi Abd_Allah

**Affiliations:** ^1^Department of Zoology, Dr. Harisingh Gour Central University, Sagar, India; ^2^Metagenomics and Secretomics Research Laboratory, Department of Botany, Dr. Harisingh Gour Central University, Sagar, India; ^3^Department of Botany and Microbiology, College of Science, King Saud University, Riyadh, Saudi Arabia; ^4^Mycology and Plant Disease Survey Department, Plant Pathology Research Institute, Agriculture Research Center, Giza, Egypt; ^5^Department of Plant Production, College of Food and Agricultural Sciences, King Saud University, Riyadh, Saudi Arabia

**Keywords:** microbes, human microbiome, host-microbe interactions, metagenomics, next generation sequencing, bioinformatics, dysbiosis, diseases

## Abstract

The interaction between the human microbiome and immune system has an effect on several human metabolic functions and impacts our well-being. Additionally, the interaction between humans and microbes can also play a key role in determining the wellness or disease status of the human body. Dysbiosis is related to a plethora of diseases, including skin, inflammatory, metabolic, and neurological disorders. A better understanding of the host-microbe interaction is essential for determining the diagnosis and appropriate treatment of these ailments. The significance of the microbiome on host health has led to the emergence of new therapeutic approaches focused on the prescribed manipulation of the host microbiome, either by removing harmful taxa or reinstating missing beneficial taxa and the functional roles they perform. Culturing large numbers of microbial taxa in the laboratory is problematic at best, if not impossible. Consequently, this makes it very difficult to comprehensively catalog the individual members comprising a specific microbiome, as well as understanding how microbial communities function and influence host-pathogen interactions. Recent advances in sequencing technologies and computational tools have allowed an increasing number of metagenomic studies to be performed. These studies have provided key insights into the human microbiome and a host of other microbial communities in other environments. In the present review, the role of the microbiome as a therapeutic agent and its significance in human health and disease is discussed. Advances in high-throughput sequencing technologies for surveying host-microbe interactions are also discussed. Additionally, the correlation between the composition of the microbiome and infectious diseases as described in previously reported studies is covered as well. Lastly, recent advances in state-of-the-art bioinformatics software, workflows, and applications for analysing metagenomic data are summarized.

## Introduction

Microbes are ubiquitous in nature, inhabiting almost every conceivable environment, and play an important role in human life. Microbes, though generally invisible, play an essential role in ecosystem functioning (1, 2), modulating key ecosystem processes such as plant growth, soil nutrient cycling, and marine biogeochemical cycling (3–6). An innumerable number of symbiotic, pathogenic, and commensal microbes colonized the human body; collectively constituting the human microbiota. Interactions between the human body and gut-microbiota are widely recognized as influencing several aspects of human health (7). A functioning microbiome is obligatory for host organisms, as it contributes to the smooth functioning of important physiological processes. In fact, host organisms have co-evolved with their microbiota; with some commensals having evolved as pathobionts while others as symbionts (8, 9). The presence of certain commensals in the human gut induces signals that drive proper functioning and maturation of the immune system. Microbial communities take on a specific structure within different hosts and physical environments (10). Consequently, identification and characterization of the microbes inhabiting a host, their distinct host phenotypes, and the biochemical pathways by which microbes impact their hosts are the major focus of host-microbiome research.

Analyses of host-microbe interactions can reveal the core characteristics of the interaction, including their identification, classification, profile prediction, and mechanisms of interaction. Although the structure, function, dynamics, and interactions of these microorganisms play an essential role in human metabolism; their identification, quantification and characterization can be problematic. The majority of microbial communities are extremely diverse and most of the individual organisms have not yet been cultured (11). Secondly, their interaction with each other and tendency to form intricate networks makes it difficult to predict their behavior (3). Establishing mechanistic connections between gut-microbiota and its functioning adds an extra challenge especially in understanding the biology of intricate microbial consortia (12). Classic approaches to microbial ecology have relied on cultivation-dependent techniques to study host-microbe interactions. Although these culture-dependent techniques have generated interesting data sets, they have also resulted in a spurious view of microbiota. Recently, however, a number of culture-independent techniques, mainly PCR-based methods, have evolved for the qualitative and quantitative identification of microbes. These techniques have entirely changed the perception of the human microbiome and have paved the way for the establishment of metagenomics. Metagenomic studies are increasing our knowledge of host-pathogenic interactions by revealing the genes that potentially allow microbes to influence their hosts in unexpected ways. Metagenomic studies of host-microbe interactions can provide useful information applicable to a wide array of disciplines; including pathogen surveillance, biotechnology, host-microbe interactions, functional dysbiosis, and evolutionary biology (13). Recent studies of host microbiomes using metagenomic approaches have offered key insights into host-microbe interactions.

**Figure 1:**
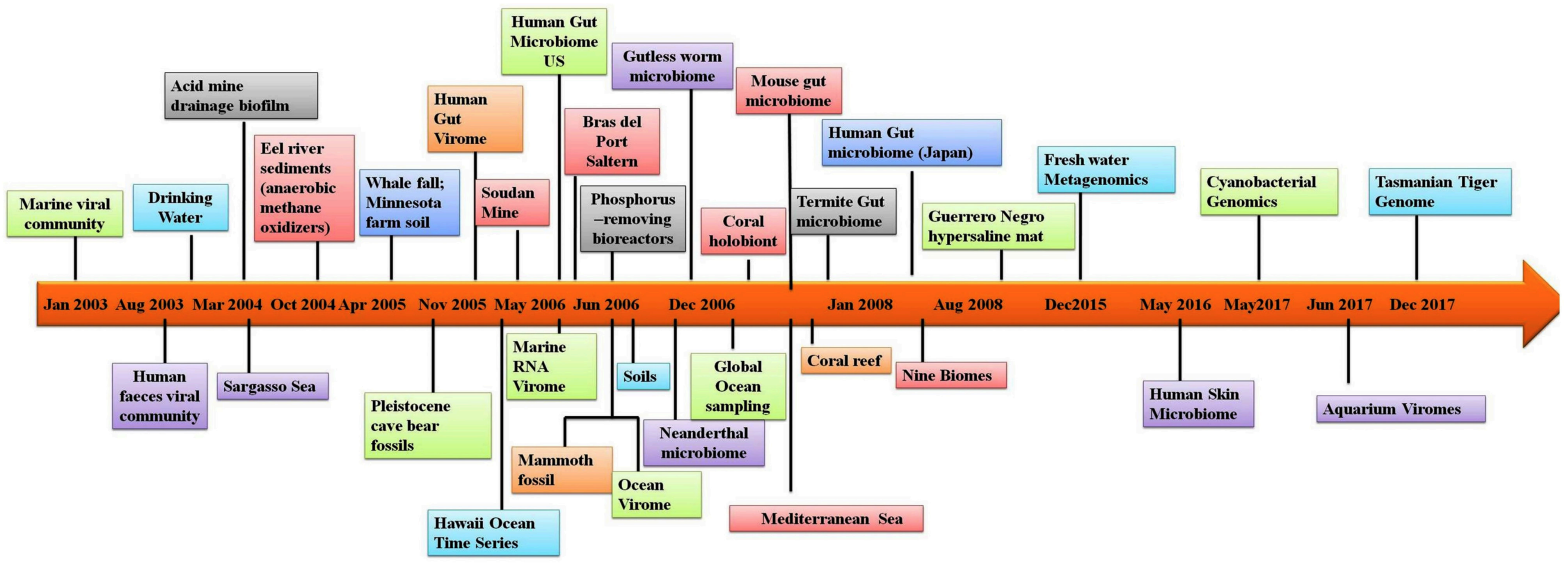
Timeline of the sequence-based metagenomic projects showing the variety of the environmental samples.

In addition to allowing researchers to characterize the composition of microbial communities, metagenomic studies have also provided novel information on other aspects of the biological sciences. For example, metagenomic studies on the human microbiome have revealed possible links between the gut microbiome and human diseases such as depression (14), rheumatoid arthritis (15) and diabetes (16). Several studies have utilized materials from ancient communities to trace changes in the microbiome. These studies have conducted metagenomic studies of coprolites (17), teeth (18), and other tissues (19). Provided that nucleic acids can be extracted from the sample, almost any material from an environment can be used in metagenomic analyses. One of the largest metagenomic studies to date is the Global Ocean Sampling. Metagenomics is also being applied to the field of medicine. Figure 1 illustrates the timeline of sequence-based metagenomic studies and shows the range of environments that have been sampled and analyzed between 2003 and 2017. Several articles have been published that have focused on metagenomic methodology and analysis software (20–27). The present review attempts to provide an overview of the high-throughput sequencing technologies and analytical software currently available for studying host-microbe interactions. Moreover, there is an attempt to also highlight the advancement of sequencing techniques over time and provide information regarding the appropriateness for applications in exploring the human microbiome and the metagenomes of other diverse environments. Lastly, a discussion is provided of the various bioinformatic options that are available to successfully meet both *de novo* sequencing and sequence alignment challenges.

## Unseen Microbial Diversity And Its Global Implications

Microbes conduct significant functions that greatly benefit the health of planet, as well as its inhabitants. Microbes help to regulate, modulate, and maintain earth's atmosphere (28), support the growth of plants and help to suppress plant diseases (29), contribute to human health (30), breakdown harmful chemicals present in contaminated environments (31, 32), support sustainable farming (33), modulate greenhouse gases (34), are primary components of various biogeochemical cycles (35) and greatly contribute to ecological processes, including climate change (36). In addition to remediating contaminated environments and modulating the atmosphere, the combined activity of these invisible microbial communities shape the face of the biosphere and represent untapped reservoirs of novel biomolecules; including pharmacological drugs and industrial enzymes (37). Microbes coexisting in the human body offer a variety of benefits by modulating fundamental metabolic processes, immunity, and signal transduction. Increasing evidence suggests that there is a significant association between the human gut microbiome and the development of human diseases (38).

Previously, it was difficult to study microbes in their natural environment and thus microbiologists were limited to studying individual species in the laboratory. This approach, however, has limited the data that can be obtained on microbial communities inhabiting diverse ecosystems. Metagenomics has helped to resolve this limitation and has greatly increased our understanding of entire microbial communities, thus significantly advancing our knowledge of microbial ecology and microbiology in general. Metagenomics, supported by next-generation-sequencing (NGS) has literally removed the limitations and boundaries associated with classic culture-based approaches (39–41). NGS technology has enabled the comprehensive study of diverse microbiomes in their native environments, including the ocean microbiome (42), human skin microbiome (43), human microbiome (44) and the Saragossa Sea microbiome (45). Some of the novel findings enabled by metagenomics involve the identification of endosymbiotic bacterial phyla (46), nitrification processes (47, 48), human disease pathogens associated with epidemics (49), bacteria (50), and viruses (51) associated with inflammatory bowel diseases, and the identification of commensal gut bacteria (52).

## Microbiome in Human Health And Disease: a Mechanistic Link

The human body serves as a host to a networked community of microorganisms (microbiome) that outnumber the body's own cells. Research on the human microbiome has been the area of immense interest over the past few years due to intimate linkage of the microbiome with human health. The human microbiome “our second genome” has intimately co-evolved with humans for millions of years and plays a critical role in human health. Deciphering the composition and function of the human microbiome can provide a deeper understanding of its' structural and functional properties. In the future, our understanding of the human microbiome and the application of metagenomic analyses will greatly enhance our understanding of human health and disease in specific individuals. The exploration of human microbiome and metagenome is considered to represent a frontier in human genetics.

The majority of research on the human microbiome has focused on the microbes colonizing the human digestive system, as these microorganisms are believed to influence human health in a number of ways. The digestive system microbiome is extremely diverse, with significant variations in its constituents across individuals (44). Modulation of the microbiome by extraneous factors, such as fecal transplantation and dietary intervention, has been shown to be a potential therapeutic approach to addressing a number of health-related problems (53). The gastrointestinal tract (GIT) harbors a vast diversity of microbes, comprising the intrinsic networks of both microbe-microbe and host-microbe interactions (54). Microbial guilds (species that exploit the same resources) have been found to provide an interesting feature that can be used to help understand processes taking place at both a single cell and community level. Microbes under normal physiological conditions are commensal and mediate digestion, strengthen the immune system and inhibit or prevent pathogens from invading the body. The linkage between the human microbiome and human health remains largely unknown and unexplored, however, a number of epidemiological studies have found that the overall reduction in the diversity of digestive system microbiota is linked to diseases such as eczema (55), asthma and inflammatory diseases (56), diabetes and obesity (57), allergies (58), digestive tract disorders such as IBD (inflammatory bowel disease) (59) and IBS (irritable bowel syndrome) (60). Dysbiosis (microbial imbalance) has also been linked with the genesis and evolution of a plethora of other diseases, including chronic fatigue syndrome (61), cancer (62), colitis (63) bacterial vaginosis (64), and anxiety and depression (65). Several recent studies have highlighted the critical role that the gut microbiome plays in modulating different immune responses, including immune tolerance, *via* Treg (T regulatory) cell modulation. Studies carried out by Geuking et al. (66), indicated that short-chain fatty acids (SCFA) can promote the development of Treg cells in the gut. Gut-inhabiting microbes facilitate the breakdown of complex carbohydrate (67) and help in the utilization of polysaccharides (68). Other examples of the health-supporting functions of the gut-microbiome are protection against diseases *via* immune modulation (69), fecal microbiome transplantation (70), metabolism, xenobiotic toxicity and pharmacokinetics (71).

## The Microbiome as a Therapeutic Agent

As mentioned, the human body is teeming with trillions of microbes, collectively called the “human microbiome.” Microbiome studies have now become a prominent field of research by offering potential and novel methods of disease diagnosis, prognosis, and treatment. Microbial ecology within an ecosystem involves a cross-talk among its inhabitants. The growth and survival of microbes in any ecosystem are largely governed by their chemical environments, and microbes have evolved the ability to adapt and utilize different chemicals through specific genes (72, 73). Alterations (good and bad) in the microbial equilibrium of the gut microbiome do occur. Science has developed medications that have a significant impact on the microbial equilibrium. Beneficial microbes colonizing the gut produce a variety of chemicals, including analgesics, vitamins, antioxidants and anti-inflammatory factors that protect and support the normal functioning of the human body. Dysbiosis (disruptions in microbiota) has been associated with different diseases. Therefore, maintaining a beneficial gut microbiome, in terms of both composition and function, is important for human health (74, 75). The gut microbiome has an active relationship with its human host and exhibits a regulatory role in cognition, mood, pain, and anxiety, exerted through a gut-brain axis. Drastic changes in the maternal microbiome that occur during pregnancy influence the maturation and immunity of neonates. Studies carried out by Ng et al. (76) indicated that increased levels of salicylic acid in the intestines contribute to the proliferation of pathogenic bacteria in the GIT when patients are treated with antibiotics. Roberfroid et al. (77) reported that the consumption of prebiotics (indigestible plant fiber) induces specific changes in the gut microbiome, elevating levels of SCFA (short chain fatty acid). Studies reported by Cani et al. (78) stated that fermentation activity carried out by the gut microbiome results in reduced hunger and increased satiety levels, which as a result, decreases total energy inputs. Similarly, studies carried out by Archer et al. (79) and Whelan et al. (80) confirmed that fermentation of non-digestible carbohydrates by the gut microbiome controls food intake activity and reduces energy intake. According to Parnell et al. (81), prebiotic-induced changes in the gut microbiome of obese patients decreases the circulation of lenomorelin or ghrelin (a hunger hormone) and increases the peptide, tyrosine or PYY. In contrast, however, studies carried out by Peters et al. (82) and Hess et al. (83) indicated that prebiotic treatments do not influence the appetite. A recent study by Tarini et al. (84) demonstrated that a single dose of insulin significantly decreases levels of lenomorelin blood plasma and augments post-prandial plasma levels of Glucagon-like peptide-1. In short, there is a growing body of evidence on the contribution of the microbiome on human health and increased understanding that the microbiome can serve as a potential therapeutic agent.

## Dissecting the Host-Pathogen Microbiome

**Figure 2:**
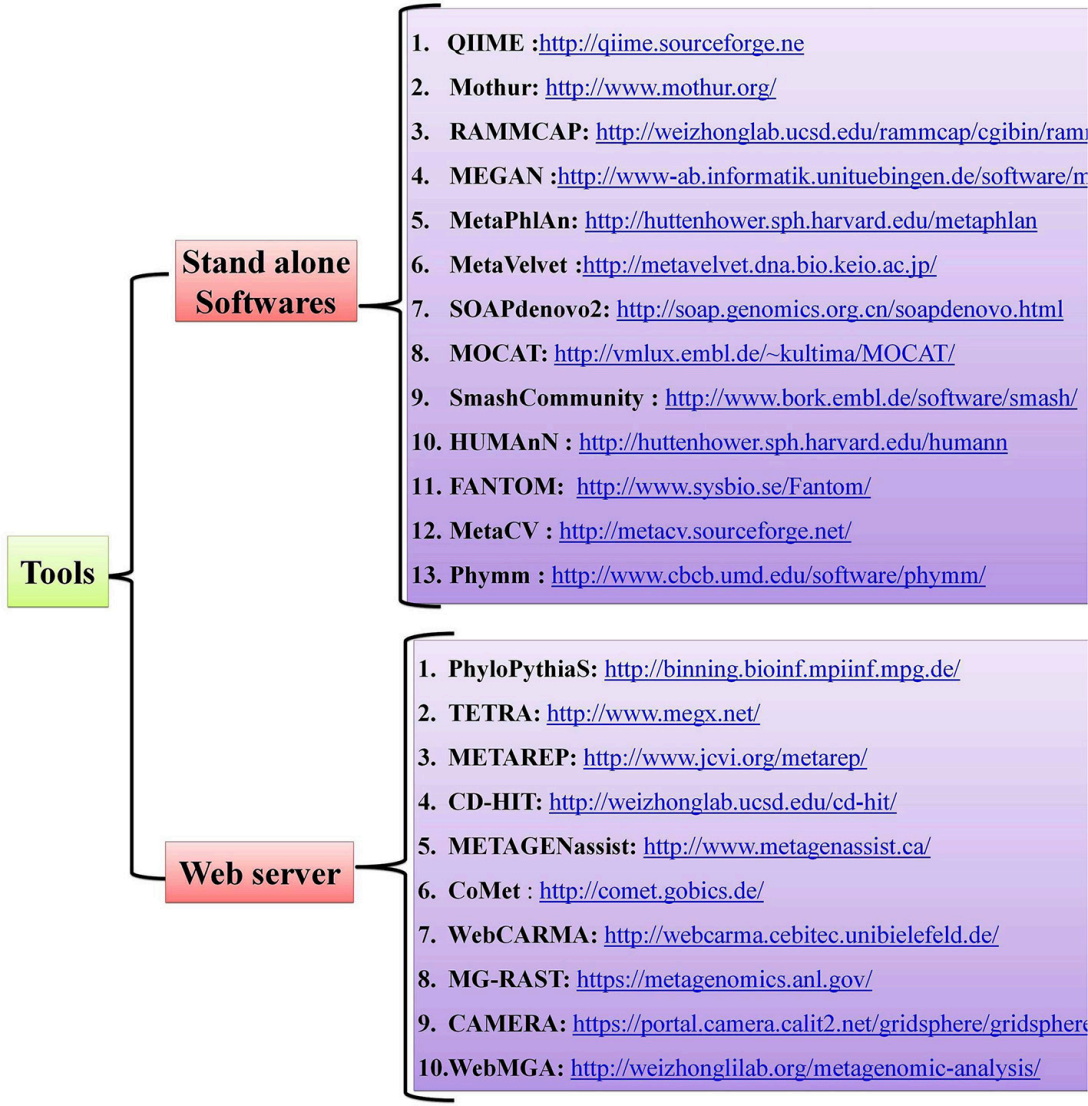
Tools and web servers related to gut microbiome studies.

Host-pathogen interactions have profound consequences in human biology and can be viewed as a battle between two systems. Pathogens, which are the invaders, can seize host cells and use them for their own advantage (8), and they can evolve so quickly that they overpower the human immune system, as with HIV infection (85). The conflict between the interacting partners results in phenotypic changes and is believed to be the main driving force for a number of phenomena, such as speciation and the evolution of sex (86). Detailed mechanistic analyses of host-pathogen interactions are varied with most still in need of further study. Notably, little is known about the molecular level dynamics of host-pathogen interactions and the need for more studies on this topic are critical, especially those dealing with the molecular events that regulate phenotypic changes in the host. Advancements in Next Generation Sequencing (NGS) technologies and bioinformatic tools have offered new approaches for studying host-pathogen systems. Researchers are now able to construct the genomes of both model and non-model organisms. The use of these newly-developed tools allows researchers to not only study the behavior of a single gene under different conditions but also study the extensive impact of these host-pathogen interactions on molecular environments (global gene expression). Several open-source, standalone R packages and web-based software programs have been developed to help and acquire key insights in understanding the host-pathogen microbiome (Figure 2). A more detailed account of metagenomic software and resources are given in a separate section of this review wherein we mentioned some of the standard software used for quality control, taxonomic classification, diversity metrics, annotation and functional information, sequence classification, metabolic pathway reconstruction, and statistical analyses.

## Microbial Census

**Figure 3:**
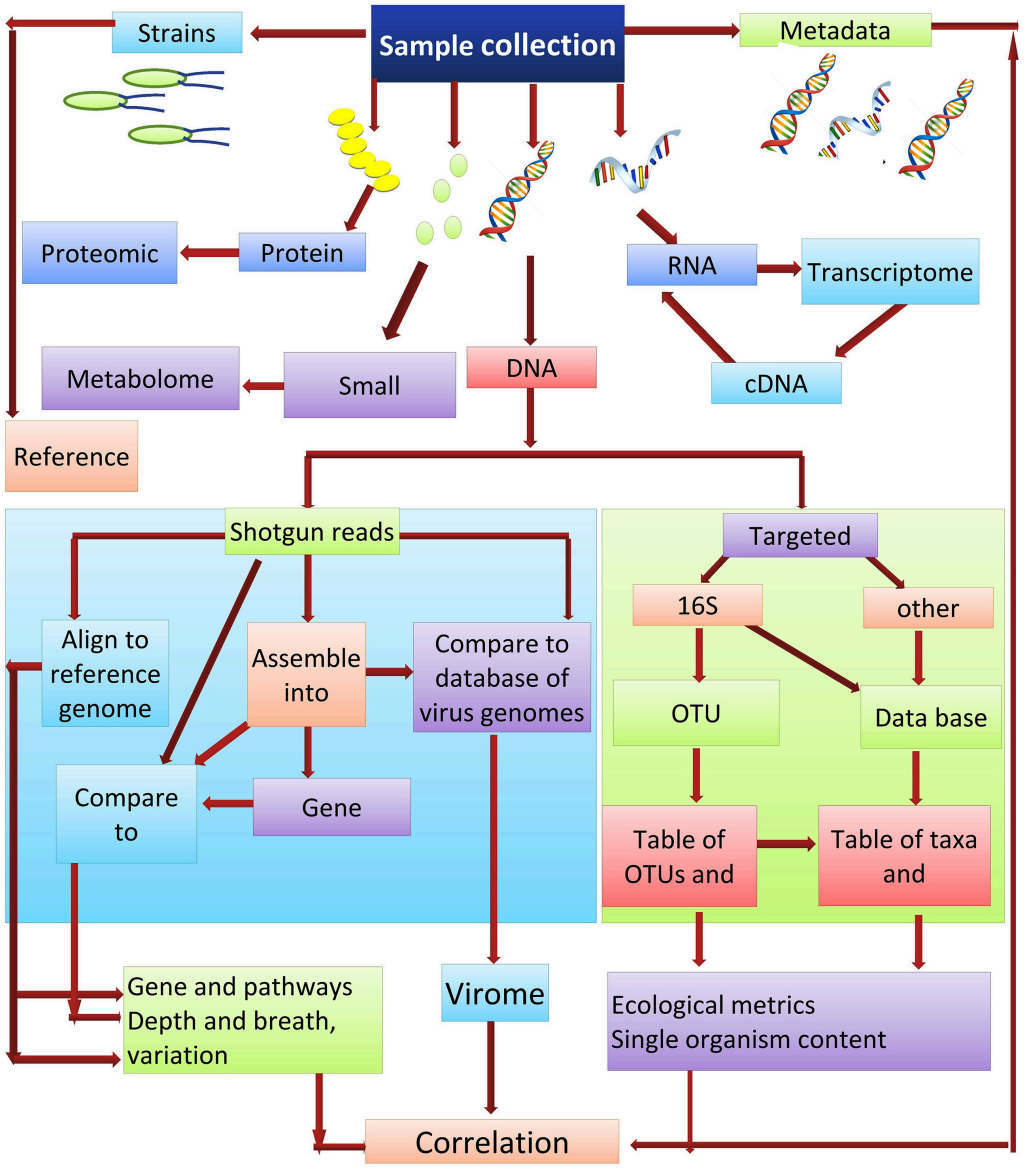
Microbiome analysis workflow.

Culture-independent methods are the most appropriate for ascertaining the abundance of microbes that are present within a community. DNA re-association kinetics provide information on both community structure and diversity (87). 16S rRNA gene sequencing is one of the main methods used for identifying the microbial taxa present in a community (88). The utility of this approach is based on the fact that the DNA sequence of regions between conserved areas of 16S rRNA vary among different bacterial species and can be species specific. Two different sequencing approaches used for studying microbial communities are (i) the targeted sequencing (16Sr RNA) and (ii) shotgun sequencing of the metagenome. Each of these methods can provide strikingly different results when used in metagenomic analyses. Shotgun sequencing methods are generally considered superior for the identification and characterization of microbial communities, as they typically provide a greater level of diversity compared to amplicon sequencing (89). Amplicon-based sequencing matches the DNA sequence amplified using a set of universal primers based on the highly-conserved 16S rRNA to sequences of known bacterial taxa. In contrast to amplicon sequencing, shotgun sequencing engages a genome-wide approach, utilizing random strings of genomic DNA sequences obtained by breaking total genomic DNA and matching the obtained sequences to an annotated database of known DNA sequences using clade-specific marker genes or common sequences. Shotgun metagenomics is often used for gene cataloging and functional inference (10). Deep sequencing of metagenomic samples, as was used in the Human Microbiome Project and Metagenomics of the Human Intestinal Tract, provides extensive sequence information even of minor components (taxa) present in the metagenome. This allows for the identification and characterization of the genes present within a given microbial community. The obtained sequences reads can either be used directly or first assembled into contigs, which are then compared to an available database for the identification of specific genes. *De novo* gene prediction is also possible (90), which may identify motifs with functional inference. Gene catalogs can also be compared with databases such as KEGG (the Kyoto Encyclopedia of Genes and Genomes) (91), which arranges the gene products into biological processes and pathways (Figure 3).

## Metagenomics and Microbial Studies

Metagenomics is expected to play a major role in advancing our understanding of microbes and microbial communities. It is tempting to suggest that metagenomics can serve as a “universal test” for pathogens, eliminating the need to perform lengthy serial testing involving specific assays. Recent advances in sequencing techniques allow almost the entire genome of individual microorganisms to be assembled directly from environmental samples. Metagenomic analyses are playing a decisive role in the characterization of human microbial communities, as well as in determining the relationship between the resident microbiome and invasive pathogens. The accumulation of sequencing data has enhanced our recognition and understanding of the changing nature of microbial populations and their impact on the environment (92) and on human health (93). Metagenomics is not only helping to identify and characterize the human gut microbiome but is also identifying novel genes and microbial pathways, as well as functional dysbiosis. Clearly, metagenomics has become an indispensable and fast-growing discipline in modern science. Advances in NGS has led to a substantial increase in the number of metagenomic studies listed in the Genomes Online Database (GOLD) (https://gold.jgi.doe.gov). These studies span a broad environmental spectrum, including natural communities; as well as engineered and clinical environments (94, 95).

## Study of Microbiome Prior To NGS

Prior to the advent of NGS technologies, the accurate profiling of microbial communities was challenging. The same was true for characterizing the human gut microbiome, a highly dense and diverse community containing only a small proportion of microbes that could be cultured (96). Early studies of the human gut microbiome involved the culturing of the microbes present in samples (97) and studying the interactions between co-cultured microbial taxa (98). These techniques, however, provided information on only a limited set of microbial taxa and microbial interactions. They failed to provide information about the composition of the entire community and the dynamics occurring between the taxa comprising the total community. The emergence of NGS technologies has overcome the limitations characteristic of studies based on culturing techniques.

## Deciphering Host-Pathogen Interactions In The Era Of NGS Technologies

The advent of NGS technologies have greatly enhanced the ability to identify and characterize metabolic and regulatory mechanisms through which hosts and microbes interact with each other to define a healthy or diseased state in the host organism. NGS technologies are invaluable for the exploration of the composition of the microbiome and exploring the genetic, functional, and metabolic properties of the microbial community. Sanger sequencing (99), the first generation of DNA sequencing technology, was one of the widely used sequencing method for more than three decades and is still used today for low-throughput DNA sequencing or sequencing of single DNA entities. Sanger DNA sequencing is based on the principle of the selective incorporation of chain-terminating dideoxynucleotides by DNA polymerase. This technique was the major approach used in the Human Genome Project in 2001. The high cost of Sanger sequencing and volume (number of sequences) limitations reduced its potential for high-throughput sequencing.

### Exploring Host-Pathogen Interactions

Advances in NGS technologies now provide a fast, cost-effective approach to delivering large volumes of highly-accurate data that has resulted in a major paradigm shift over the past few decades (100, 101). Time and cost were originally the main stumbling blocks associated with sequencing technology. The advantages of NGS over classic Sanger sequencing are that it is cost-effective, devoid of a cloning step, offers highthroughput, and requires minimal technical expertise. A major challenge with NGS data, however, is the analysis of millions of sequences that allows one to achieve statistically and scientifically meaningful conclusions (Table 1).

**Table 1:** Advantages and limitations of available Next generation sequencing (NGS) platforms.

**Sequencing reaction**	**Limitation**	**Advantages**	**Instruments**	**Read length in base pairs (bp)**	**Throughput**	**Total number of reads**	**Runtime**
Sequencing by ligation or SOLiD sequencing 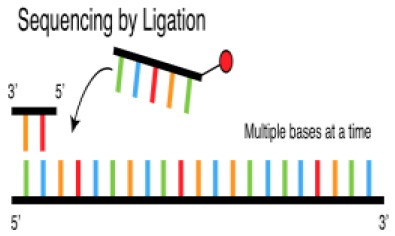	This sequencing method has been reported to have problems in sequencing particularly palindromic sequences and relatively slower than other methods.	Relatively cheap	SOLiD 5500 Wildfire	50 (SES)	80 Gb	~700 M^+^	6 days
				75 (SES)	120 Gb		
				50 (SES)^+^	160 Gb		
			SOLiD 5500xl	50 (SES)	160 Gb	~1.4 bn^+^	10 days^+^
				75 (SES)	240 Gb		
				50 (SES)^+^	320 Gb		
			BGISEQ-500 FCS155^*^	50–100 (SES/PES)^+^	8–40 Gb^+^	NA^†^	24 h
			BGISEQ-500 FCL155	50–100 (SES/PES)^+^	40–200 Gb^*^	NA^†^	24 h^+^
Sequencing by synthesis:CRT 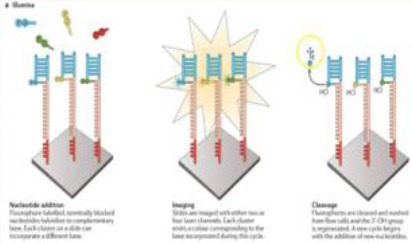	Equipment are very expensive. Requires high concentration of DNA.	Potential for high sequencing yield, depending upon sequencer model and desired application	Illumina MiniSeq Mid Output	150 (SES)^+^	2.1–2.4 Gb^+^	14–16 M^+^	17 h^+^
			Illumina MiniSeq High output	75 (SES)	1.6–1.8 Gb	22–25 M(SES)^+^	7 h
				75 (PES)	3.3–3.7 Gb	44– 50 M(PES)^+^	13 h
				150 (PES)^+^	6.6–7.5 Gb^+^		24 h^+^
			Illumina MiSeq v2	36 (SES)	540–610 Mb	12–15M (SES)	4 h
				25 (PES)	750–850 Mb	24–30 M (PES)^+^	5.5 h
				150 (PES)	4.5–5.1 Gb		24 h
				250 (PES)^*^	7.5–8.5 Gb^+^		39 h
			Illumina MiSeq v3	75 (PES)	3.3–3.8 Gb	44–50 M (PES)^+^	21–56 h^+^
				300 (PES)^+^	13.2–15 Gb^+^	
			Illumina NextSeq 500/550 Mid output	75 (PES)	16–20 Gb	Up to 260 M (PES)^+^	15 h
				150 (PES)^+^	32–40 Gb^+^		26 h^+^
			Illumina NextSeq 500/550 High output	75 (SES)	25–30 Gb	400 M(SES)^+^	11 h
				75 (PES)	50–60 Gb	800 M(PES)^+^	18 h
				150 (PES)^+^	100–120 Gb^+^		29 h^+^
			Illumina HiSeq2500v2 Rapid run	36 (SES)	9–11 Gb	300 M(SES)^+^	7 h
				50 (PES)	25–30 Gb	600 M(PES)^+^	16 h
				100 (PES)	50–60 Gb		27 h
				150 (PES)	75–90 Gb		40 h
				250 (PES)^+^	125–150 Gb^+^		60 h^+^
			Illumina HiSeq2500 v3	36 (SES)	47–52 Gb	1.5 bn (SE)	2 days
				50 (PES)	135–150 Gb	3 bn(PES)^+^	5.5 days
				100 (PES)+	270–300 Gb		11 days^+^
			Illumina HiSeq2500 v4	36 (SES)	64–72 Gb	2 bbn(SES)	29 h
				50 (PES)	180–200 Gb	4 B (PES)^+^	2.5 days
				100 (PES)	360–400 Gb		5 days
				125 (PES)^+^	450–500 Gb^+^		6 days
			Illumina HiSeq 3000/4000	50 (SES)	105–125 Gb	2.5 bn (SES)^+^	1–3.5 days^+^
				75 (PES)	325–375 Gb	
				150 (PES)^+^	650–750 Gb^+^	
			Illumina HiseqX	150 (PES)^+^	800–900 Gb per flow cell^*^	2.6–3 bn (PES)^+^	< 3 days^+^
			Qiagen Gene Reader	NA^†^	12 genes; 1,250 mutations	NA^†^	Several days
Sequencing by synthesis: SBS 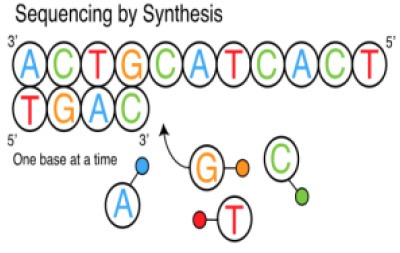	Homopolymer errors	Less expensive and relatively fast	454 GS Junior	Upto 600;400 average (SES,PES)^*^	35 Mb^+^	~ 0.1 M^+^	10 h^+^
			454 GS Junior+	Upto 1,000;700 average (SES,PES)^+^	70 Mb^+^	~ 0.1 M^+^	18 h^+^
			454GSFLX TitaniumXLR70	Upto 600;450 mode (SES,PES)^+^	450 Mb^+^	~1 M^*^	10 h^+^
			454 GS FLX Titanium XL^+^	Up to 1,000; 700 mode (SE, PE)^+^	700 Mb^+^	~1 M^+^	23 h^+^
			Ion PGM 314	200 (SES)	30–50	400,000–	23 h
				400 (SES)	60–100 Mb^+^	550,000^+^	3.7 h^+^
			Ion PGM 316	200 (SES)	300–500 Mb	2–3 M^+^	3 h
				400 (SES)^+^	600 Mb−1 Gb^+^		4.9 h^+^
			Ion PGM 318	200 (SES)	600 Mb−1 Gb	4–5.5 M^+^	4 h
				400 (SES)^+^	1–2 Gb^+^		7.3 h^+^
			Ion Proton	Up to 200 (SES)	Up to 10 Gb^+^	60–80 M^+^	2–4 h^+^
			Ion S5 520	200 (SES)	600 Mb−1 Gb	3–5 M^+^	2.5 h
				400 (SES)^+^	1–2 Gb^+^		4 h^+^
			Ion S5 530	200 (SES)	3–4 Gb	15–20 M^+^	2.5 h
				400 (SES)^+^	6–8 Gb^+^		4 h^+^
			Ion S5 540	200 (SES)^+^	10–15 Gb^+^	60–80 M^+^	2.5 h^+^
Single-moleculereal-time long reads or (PacificBioSciences) 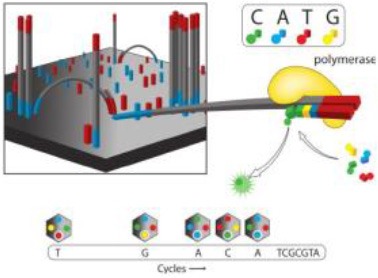	Moderate throughput and equipment are very expensive	Fast detection	Pacific BioSciences RSII	~20 Kb	500 Mb−1 Gb^+^	~55,000^+^	4 h^+^
			Pacific BioSciences Sequel	8–12 Kb	3.5–7 Gb^+^	~350,000^+^	0.5–6 h^+^
			Oxford Nanopore MK1MinION	Up to 200 Kb	Up to 1.5 Gb	>100,000	Up to 48 h
			Oxford Nanopore PromethION	NA^†^	Upto 4 Tb^+^	NA^†^	NA^†^

+ *Manufacturer's data*;

* *Rounded from Field Guide to next-generation DNA sequencers and 2014 update*;

† *Information is not available, as this product has been developed recently*.

*CRT, Cyclic Reversible Termination; NA, Not Available; PES, Paired End Sequencing; SBS, Sequencing by Synthesis; SES, Single End Sequencing*.

**Figure 4:**
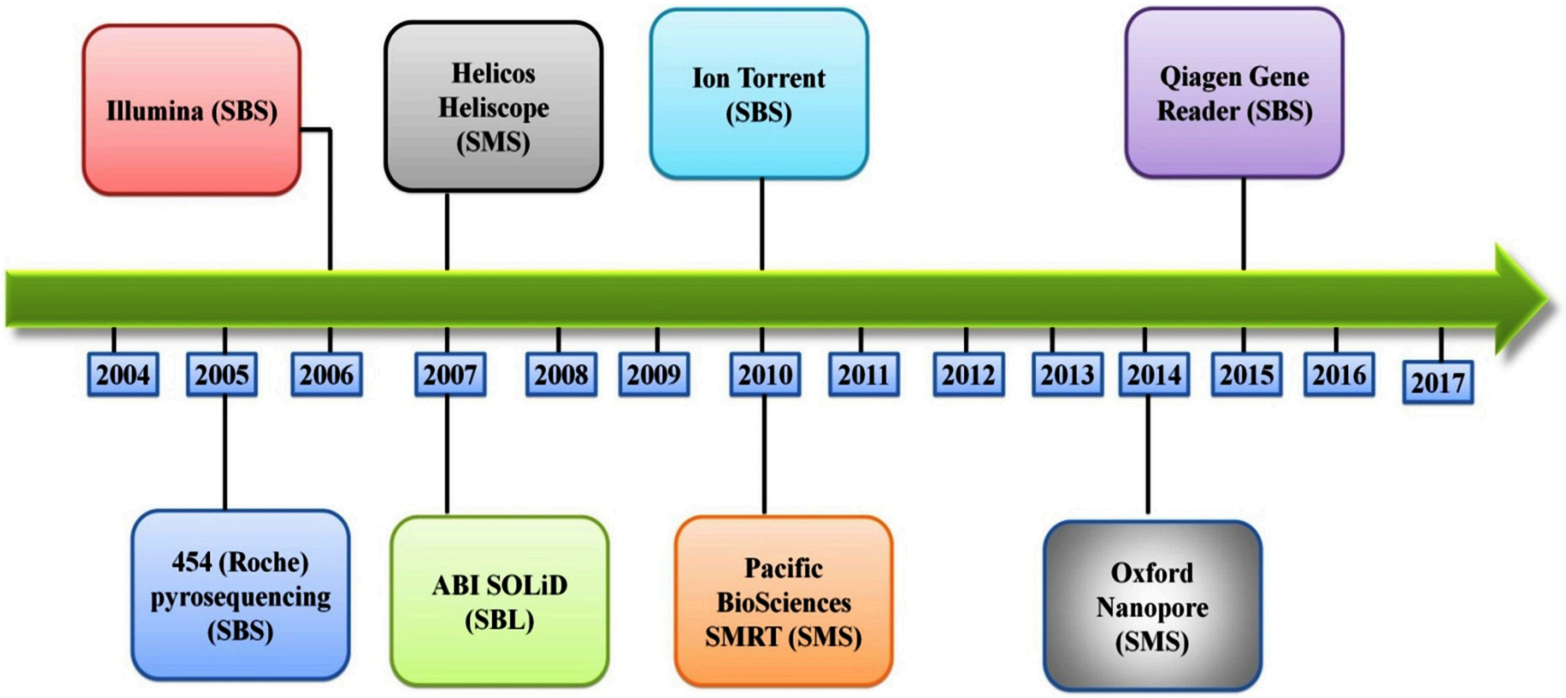
Timeline of the introduction of the next-generation DNA sequencing technologies and platforms.

Several different NGS platforms have been developed (Figure 4) and are commonly used. These include the Roche 454 GS FLX, Illumina (MiSeq and HiSeq), Ion Torrent/IonProton/Ion Proton, SOLiD 5500 series, and Oxford Nanopore. At present, the majority of microbial studies using high-throughput sequencing have focused on either functional metagenomics (103) or amplicon sequencing (104).

### Roche 454 Genome Sequencer

This sequencing platform is based on the principle of pyrophosphate release, which was originally described by Nyrén et al. (105) in 1985 and reported by Hyman (106) in 1988. Roche 454 was produced and made commercially available in 2005, and advertised as the first available high-throughput sequencing system. The system utilizes sequencing by synthesis (SBS), in which adapters are ligated to DNA fragments that cause the binding of the fragments to microbeads in a Pico Titer Plate (https://www.roche.com/). Amplification of the DNA fragments is carried out by Emulsion PCR, in which water droplets containing a single bead and PCR reagents are immersed in oil. The long read length (400–500 bases with paired-ends), along with its high efficiency, were more advantageous than what other NGS platforms could provide at that time; and thus was used for genome sequencing. The system generates 20 Mb of sequences per run with an average read length of approximately 100 bp (107). One of the notable applications of the Roche 454 system was the identification of the agents responsible for the epidemic disease of honeybees (108). Additional information about Roche 454 Genome Sequencer can be obtained at http://www.roche.com.

### Illumina Genome Sequencer

The Illumina sequencing platform first emerged in 2006, and was followed by the acquisition of Solexa by Illumina in 2007. Illumina possesses an array of the most commonly sequencing systems and has rapidly been adopted by many researchers throughout the world. This is due to its' cost-effectiveness, and longer read length (although a limitation in the earlier version of the Illumina, which was subsequently improved in the newer version, MiSeq 2 × 300 bp). This led to a major shift by the scientific community from using the Roche 454 platform to Illumina technology (109). Illumina follows the principle of SBS chemistry, by incorporating reversible chain terminator nucleotides for all four bases, the labeling of each base with a different fluorescent dye, and the use of a DNA polymerase (110). Sequencing involves the ligation of specific adapters to both ends of short DNA fragments, and the immobilization of one of the adapters by binding to a solid support. The adapters hybridize with specific oligonucleotides bound to a proprietary substrate within a micro fluid flow cell. Fluorophore-bearing nucleotides are then introduced one by one and incorporated into the growing complementary strand by a DNA polymerase. Sequential images are captured and analyzed to identify the nucleotide that is incorporated in the growing strand and the cycle is repeated with different nucleotide species. The resulting reads have a final length of 35 nucleotides (111).

Illumina, however, introduced an upgraded version of their technology, the Genome Analyser II, which tripled the output relative to earlier versions of the Genome Analyser. Presently, the IlluminaMiSeq offers one of the longest (300 bp) read lengths of all of the Illumina products; facilitating the sequencing of paired-end reads (104). Another Illumina platform, the Illumina HiSeq, however, is able to generate approximately 200 Gbp of sequences with a single read of 2 × 100 bp (paired-end) per run (112). Additional information about the various Illumina sequencers can be obtained at http://solexaqa.sourceforge.net/ (113).

### Qiagen Gene Reader

In 2012, Qiagen introduced the Intelligent BioSystems cyclic reversible termination platform, which was commercialized in 2015 under the name Gene Reader (114). In contrast to other next-generation platforms, the Qiagen Gene Reader is the first all-in-one platform that can execute all of the steps required for sequencing DNA, from sample preparation to analysis. To achieve this goal, the Gene Reader sequencer was combined with the QIA cube sample preparation system and the Qiagen Clinical Insight platform for variant analysis. Gene Reader virtually utilizes the same approach as Illumina, apart from the fact that only a small fraction of the added nucleotides incorporate fluorophore-labeled dNTP (115). Qiagen's Gene Reader usually runs up to four flow cells at a time, with each flow cell running up to ten samples. The flow cells can be added in mid-run via a “turntable” within the instrument. Additional information on the Qiagen Gene reader can be obtained at http://www.qiagen.com.

### ABI SOLiD (Sequencing by Oligonucleotide Ligation and Detection) System

Applied Biosystems, through the Life Technology subsidiary, introduced the SOLiD platform in 2007. The system employs a unique chemistry for sequencing by ligating oligonucleotide adapters to DNA fragments and immobilizing the ligation products on beads, which are then placed on a water-oil emulsion (116). The beads on which DNA amplification occurs are deposited on glass slides and subjected to sequential hybridization with a universal PCR primer complementary to the adapters. The ligation step is then followed by fluorescence detection.

### Ion Torrent Sequencing Technology (PGM, Proton, S5 Series)

Ion Torrent introduced the personal genome machine (PGM) in 2010 as a cost effective platform for DNA sequencing (117). Unlike other sequencing technology, Ion Torrent does not make use of optical signals (118) but rather utilizes an enzymatic cascade to generate a signal. The Ion Torrent system utilizes high-density micro-machined wells to carry out nucleotide additions in a massively parallel approach. Each micro-well contains a different DNA template. There is an ion-sensitive layer and an ion-sensor located under each well. The technology works on the principle of detecting the proton (H^+^) released during the incorporation of each dNTP in a growing DNA template. The release of H^+^ ion results in a change in pH that is detected by an integrated ion-sensitive, field-effect transistor (ISFET) (117). In the case of two identical bases, the output voltage is doubled. Ion torrent platform can generate upto 10 Gb of sequence data in a single run, with a maximum of 50 million reads having an average read length of 200 bases. The PGM can also provide 5.5 million reads having an average read length of 400 bp, producing a maximum of 2 Gb of sequence data from 318 V2 chip. A notable aspect of this technology is the size-selection step in which sequencing of longer fragments is omitted (https://www.thermofisher.com/in/en/home/brands/ion-torrent.html). Additional information about Ion Torrent technology can be obtained from https://www.thermofisher.com/in/en/home/brands/ion-torrent.html.

## The Third Generation of Sequencing Technology

At present, the described sequencing technologies are the most commonly used for metagenome projects, however, sequencing technologies have undergone rapid advances during the past few years to attempt to resolve the biases associated with the current methods and to obtain a better balance between data yield, read length, and cost. These efforts have resulted in third generation sequencing technologies, such as Oxford Nanopore (119), and PacBiosequencing platforms (120) which are single-molecule and real-time technologies that reduce amplification bias, as well as short read length problems. The reduction in the cost and time presented by these sequencing methods are valuable asset. Although the error rate with the newer technologies is much higher relative to the other described sequencing technologies, this problem can be addressed by increasing the sequencing depth.

### Pacific Biosciences

Pacific Biosciences established the first DNA sequencer that utilizes a single-molecule, real-time sequencing (SMRT) approach. This sequencing platform has become one of the most widely used third-generation sequencing technologies (121). The platform is based on the sequencing by synthesis principle. Pacific Biosciences makes use of the same fluorescent dyes as other NGS technologies, however, instead of carrying out the cycles of nucleotide amplification in the same manner as other sequencing technologies, the signals emitted upon the incorporation of the nucleotides are detected in real time. Sequencing is carried out on a chip (SMRT cell) that contains several zero mode wave (ZMW) guides. A single DNA polymerase is immobilized to the bottom of each ZMW guide with a molecule of single stranded DNA template (122). Four phospholinked nucleotides, each labeled with a different fluorescent dye producing a distinct emission spectrum, are also added to SMRT cells. Once the nucleotide is incorporated by the DNA polymerase, a light signal is produced and a base call is made and recorded (122).

### Helicos Biosciences

Heliscope was released by Helicos Biosciences in 2007. It is also a single-molecule sequencing device. Sequencing is carried out in a glass flow cell with 25 channels for samples. The samples can either be replicates of the same sample or different samples. The Heliscope platform utilizes emulsion PCR amplification of DNA fragments in order to obtain significantly higher signals for reliable base detection by multiple charge-coupled device cameras. Single-molecule sequencing methods have the potential to deliver consistently low error rates by eliminating amplification-related bias, intensity averaging, and synchronization problems (123, 124). In the Heliscope platform, 100–200 oligonucleotide fragments are initially immobilized on a proprietary substrate within a microfluidics flow cell. Fluorescence-labeled nucleotides are then introduced individually and are incorporated by DNA polymerase into the growing complementary strand. The fluorophore-bearing nucleotide increases detectability and eliminates the need for amplification of the DNA template. Images are recorded and analyzed to identify the nucleotide that has been incorporated into the growing strand before the cycle begins with a different fluorescently-labeled nucleotide. At present, the Heliscope can only provide a read length of 35 nucleotides (111). Additional information can be obtained at http://www.helicosbio.com.

### Oxford Nanopore Sequencing

Oxford Nanopore Technologies (ONT) is at the forefront of developing nanopore sequencing technology (http://www.nanoporetech.com/). The Nanopore platform does not require an amplification step as a part of library preparation. The novelty of this approach is that the DNA strand to be sequenced can be directly analyzed. Oxford Nanopore Technologies introduced the MinION (125) device in 2014. It has the potential to provide longer reads with better resolution of repeated sequence elements and structural genomic variants (126). MinION is a mobile, single-molecule Nanopore sequencer measuring four inches in length and is connected to a laptop with USB 3.0. Nanopore sequencing technology is based on the principle of modulation of the ionic current as a DNA molecule traverses through the nanopore, revealing characteristics of the molecule such as conformation, length and diameter. The pore consists of a protein within a conductive electrolytic solution which creates a small potential gradient across the protein pore (127). MinION mk1B is a pocket-sized portable sequencing device, containing 512 nanopore channels, and can be directly linked to a computer for data collection. More recently, a more advanced device, “PromethION,” has been commercialized (127). PromethION is a benchtop sequencer possessing 48 individual flow cells, each consisting of 3,000 pores that are equivalent to 48 MinIONs processing 500 bp/s (128). The capabilities of this instrument provide sequencing power that is sufficient to conduct sequencing of large genomes, such as the human genome. Additional information on Oxford Nanopore sequencing technologies can be obtained at https://www.nanoporetech.com.

So far, the present review has provided an overview of the first through third generations of sequencing technology that have provided significant improvements in the ability to conduct microbiome research. Metagenomic and other omic approaches are the most effective methods that can be used to characterize microbial communities, as well as their metabolic activity. It is now feasible to obtain information on the composition (taxa), diversity, pathogenesis, evolution, and drug resistance of microbes. The selection of any of the above mentioned platforms, however, should be mainly dependent on the aim, design, and purpose of the study. Illumina sequencing technology has made tremendous advances in data output and cost efficiency over the past few years and as a result, presently dominates the NGS market (129, 130). Illumina sequencing technology has been used extensively in numerous microbiome research projects (131–133), including the Human Microbiome Project (44). While both Ion Torrent and Illumina sequencers provide a number of advantages in terms of their cost and efficiency, the short read lengths they provide make them less appropriate for addressing a number of scientific questions, including detection of gene isoforms, methylation detection, and genome assembly (118). SMRT (single-molecule real-time) sequencing platforms offer approaches that are more suited for these research objectives. Since PacBiosequencing generates longer reads that provide longer scaffolds (134–136), it is well suited for *denovo* genome assembly. The commercial availability of MinION sequencers by Oxford Nanopore Technologies, which resemble a USB flash drive in appearance, has also enabled applications that require long-read sequencing (137, 138). The efficiency, long read lengths, and single-base sensitivity make nanopore sequencing technology a promising approach for high-throughput sequencing. The MinION system has been used for sequencing the genomes of infectious agents, including the analysis of bacterial antibiotic resistance islands (137), the influenza virus (139) and genome surveillance of the Ebola virus (140). The advancements in high-throughput sequencing technologies now provide the opportunity to choose different sequencing platforms to conduct microbiome research. In a comparative analysis of the Illumina MiSeq, Ion Torrent PGM, and 454 GS Junior sequencing platforms, Loman et al. (141) reported that Illumina provided the highest output per run (1.6Gb/run, 60Mb/h) and the lowest error rates. In a study comparing different sequencing platforms (Ion Torrent PGM, Illumina MiSeq and HiSeq) for the shotgun sequencing of six human stool samples, Clooney et al. (142) concluded that the best assembly values were obtained using the Illumina HiSeq platform, in which 10 million reads per sample were produced. In contrast, the Illumina MiSeq and Ion Torrent PGM did not produce a sufficient number of reads to produce an adequate genome assembly (143).

## Correlation Between the Microbiome and Infectious Diseases

Human gut microbiome signatures exhibit individual specificity. There is a high degree of inter individual variation that is based on both host genetics and environmental factors (144, 145). The high degree of individual specificity, however, has hampered our understanding of function of the gut microbiome and its importance in health and disease. The human gut microbiome exhibits a high degree of plasticity, mainly in response to dietary changes that support a healthy gut ecosystem and minimize disease risk (146). The onset of new methodologies, including NGS and bioinformatic pipelines, have resulted in a paradigm shift in the fields of clinical microbiology and infectious diseases due to the realization of the complex interactions that occur within the microbiome. The relationship between human pathogens, infectious diseases, and the gut microbiome are slowly being revealed. Several studies have examined the correlation between the human gut microbiome and health status (141, 142). Reports have indicated that while the gut microbiome appears to be relatively stable under healthy conditions, any qualitative or quantitative changes in the gut microbiome can result in functional modifications and disease as reported (144, 147–149). A rich level of bacterial diversity is considered to be an indicator of a healthy status, while a low level of bacterial diversity is correlated with inflammatory, immune, and obesity-related diseases (58, 144, 147–153). Several studies have indicated that the human microbiota plays a crucial role in human health and disease (68, 154–168). Studies have also revealed that microbial symbiosis plays a central role in the development of a number of diseases, including liver diseases (156), metabolic disorders (154), gastrointestinal (GI) malignancy (157), respiratory diseases (158), autoimmune diseases (160), and mental or psychological diseases (160). Johnson et al. (169) discussed the *Bacteroidetes*, one of main components of the microbiome, their genetic variability and contrasting effect on metabolic diseases such as obesity and type II diabetes (169). Yiu et al. (170) proposed that body weight, metabolism, and diseases such as obesity are affected by the interplay between the immune system, metabolism and microbiome (170). In discussing chronic IBD, Frick and Wehkamp (171) outlined some of the available therapeutic interventions that can be used to alter mucosal immunity and the composition of the microbiome. While studying the molecular aspects of human gut-brain interactions, Lee et al. (172) demonstrated how the microbiota influences host physiology and neurodegenerative and neurological developmental diseases (172).

## Bioinformatic Pipelines for Metagenomic Data Analysis

**Figure 5:**
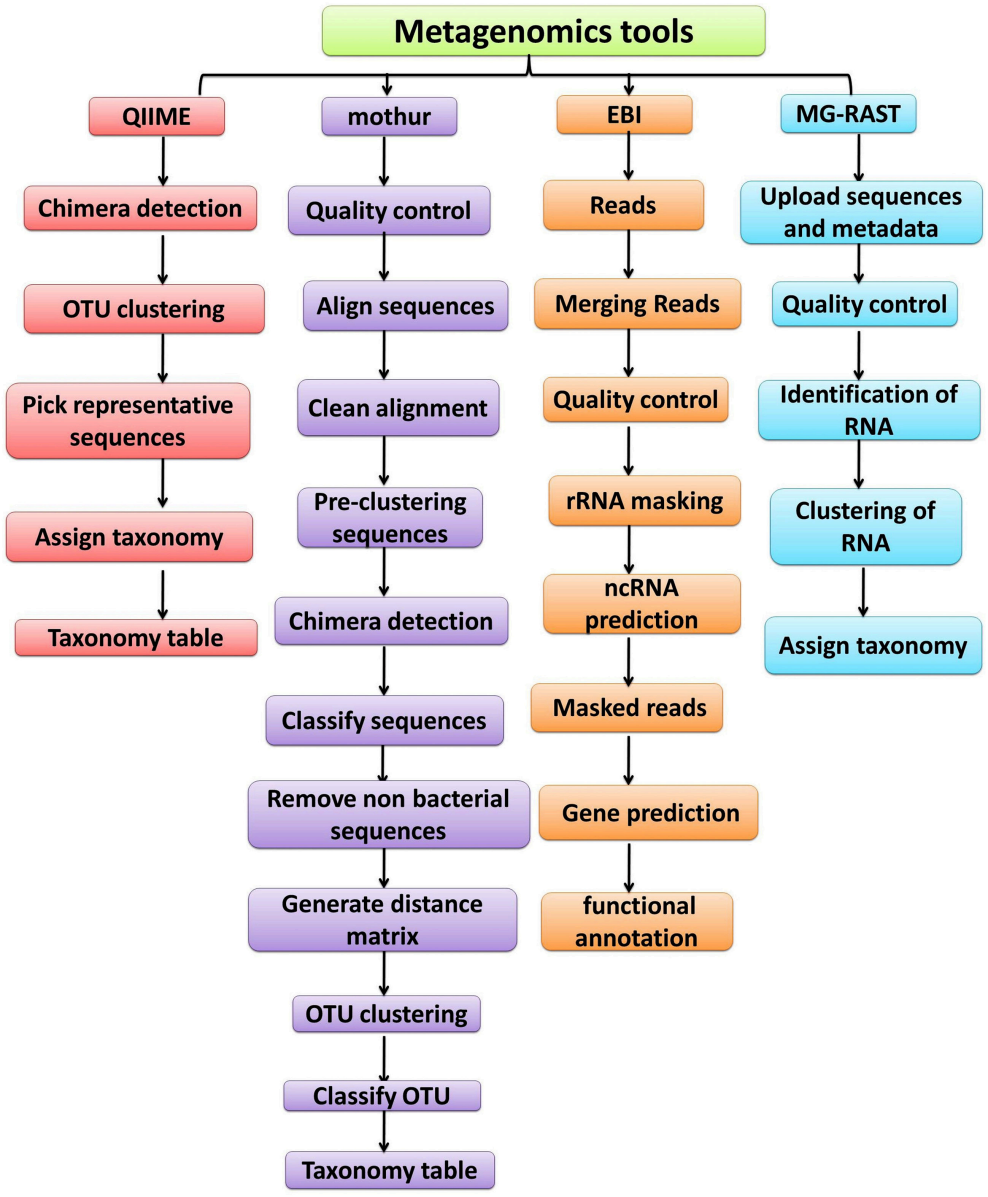
Overview of the workflow used by metagenomic analysis tools (QIIME, Mothur, EBI and MG-RAST).

The advances in NGS have resulted in the production of massive datasets that are increasingly difficult to analyse (128). As larger datasets are generated, more sophisticated computational resources and bioinformatic tools are required. The interpretation and understanding of metagenomic studies depend on the computational tools that can be used to analyse enormous data sets and mine valuable, useful, and valid information regarding the microbial communities being studied. Bioinformatic tools used for metagenomic analysis, especially for translating raw sequences into meaningful data, are continually developing with the aim of providing the ability to examine both the taxonomic and the functional composition of diverse metagenomes (173, 174). A number of the specialized software programs available for analysing the metagenomic data are listed (Table 2). Based on the list provided, an example of a comparative analysis pipeline is presented in the present review that takes into consideration user friendliness, ease of access, open source availability, ability to analyse metagenomic datasets, and ability to provide graphical representations of the analyzed data (Figure 5). A description of the software (MG-RAST, EBI, QIIME and Mothur) used in the different pipelines is described in Table 3, which provides a detailed summary of the functionality and features of the mentioned software programs. The four pipelines share several steps during the analysis such as quality control, clustering, and annotation (Figure 5).

**Table 2:** Lists of software's used in metagenomics analysis.

**Software**	**Application**	**Link (website)**	**References**
FastQC	FastQC, a java based application is performed via a series of analysis modules. FastQC can either run in a non-interactive mode or in a standalone interactive mode. FastQC is a quality control tool used for high-throughput sequence data via a series of modular options and giving graphical results of length distribution, quality per base sequence, N numbers, GC content, over representation and duplication.	http://www.bioinformatics.babraham.ac.uk/projects/fastqc/	(175)
Fastx-Toolkit	Fastx is a command based tool kit for the quality control of short-reads and allows processing, format conversion, collapsing and cutting on the basis of sequence identity and length.	http://hannonlab.cshl.edu/fastx_toolkit/index.html	(176)
PRINSEQ	A standalone tool allows integration and analysis into the existing data processing pipelines. PRINSEQ as a tool offers a computational resource that is able to handle huge amount of data generated by next-generation sequencers. It is used for sequences trimming based on in the di-nucleotides occurrences and the sequence duplication (mainly 5′/3′).	http://prinseq.sourceforge.net/	(177)
NGS QC Toolkit	NGS QC Toolkit encompasses user-friendly standalone tools for the quality control of the sequence data generated by next-generation sequencing platforms. The analysis is performed in a parallel environment.	http://www.nipgr.res.in/ngsqctoolkit.html	(178)
Meta-QC-Chain	Meta-QC-Chain is a tool for the quality control analysis performed in parallel environment. Performs mapping against 18S rRNA databases in order to remove the eukaryotic contaminant sequences.	http://www.computationalbioenergy.org/qc- chain.html	(179)
Mothur	Mothur is an open-source, expandable software used for the quality analysis of reads to taxonomic classification, ribosomal gene meta-profiling comparison and calculus of diversity estimators.	http://www.mothur.org/	(180)
QIIME	QIIME pipeline is designed for the task of analyzing microbial communities sampled via a marker gene (16S or 18S rRNA) amplicon sequencing. In its heart pipeline QIIME performs quality pre-treatment of raw-reads, calculate estimates diversity estimates, taxonomic annotation and comparison of metagenomic data.	http://qiime.org/	(181)
MEGAN	MEGAN is a graphical interface tool that allows both taxonomic as well as functional analysis of metagenomic reads. It is based on the BLAST output of short reads and performs comparative metagenomics.	http://ab.inf.uni-tuebingen.de/software/megan/	(20)
CARMA	CARMA provides a clear quantitative and statistical characterization of phylogenetic classification of the reads based on Pfam conserved domains.	http://omictools.com/carma-s1021.html	(182)
PICRUSt	PICRUSt is a tool that serves in the field of metagenomic analysis where the prediction of the metabolic potential is done from the taxonomic information obtained *via* 16S rRNA meta-profiling projects. PICRUSt could be thought of as an automated substitute to manually mining the gene families that are believed to be present in organisms whose sequences are found in a 16S ribosomal RNA.	http://picrust.github.io/picrust/	(183)
TETRA	TERTA is a web-based stand alone program used for the Taxonomic classification and comparison of tetra nucleotide patterns with in a DNA sequence.	http://www.megx.net/tetra	(184)
PhylophytiaS	Composition-based classifier of sequences based on reference genomes signatures	https://omictools.com/pps-tool	(185)
MOCAT	MOCAT is a highly configurable and modular pipeline that includes the quality treatment of metagenomic reads based on single copy marker genes classification and gene-coding prediction. The pipeline makes use of a state-of-the-art program to map quality control and assemble reads from metagenome samples sequenced at a very high depth (several billion base pairs).	http://www.bork.embl.de/mocat/	(186)
Parallel-meta	Parallel-meta is a comprehensive and automotive software package that offers fast data mining and metabolic function across large number of metagenomic datasets. The functional annotation is based on BLAST best hit results.	http://www.computationalbioenergy.org/parallel-meta.html	(187)
MetaclusterTA	MetaclusterTA is a tool used for the Taxonomic annotation that is based on the binning of reads and contigs. Dependent on reference genomes.	http://i.cs.hku.hk/~alse/MetaCluster/	(188)
MaxBin	MaxBin software is used for the unsupervised binning of metagenomic sequences based on an Expectation-maximization algorithm. For user's expediency MaxBin reports genome-related statistics including GC content, genome size and completeness.	http://bowtie-bio.sourceforge.net/index.shtml	(189)
Amphora and Amphora2	Amphora and Amphora2 is used for the Metagenomic phylotyping via single copy phylogenetic marker genes classification.	http://pitgroup.org/amphoranet/	(102, 190)
BWA	BWA is an algorithm used for the mapping of short-low-divergent sequences to large references. It is based on Burrows–Wheeler transform.	http://bio-bwa.sourceforge.net/	(191)
Bowtie	Bowtie is a fast short read aligner to long reference sequences based on Burrows–Wheeler transform.	http://bowtiebio.sourceforge.net/index.shtml	(192)
Genometa	Genometa is a graphical interface applied for taxonomic and functional annotation of short-reads metagenomic data.	http://genomics1.mhhannover.de/genometa/	(193)
SOrt-ITEMS	SOrt-Items is a tool used for taxonomic annotation via alignment-based orthology of metagenomic reads.	https://omictools.com/sort-items-tool	(194)
DiScRIBinATE	Taxonomic assignment by BLASTx best hits classification of reads.	https://www.westgrid.ca/support/software/discribinate	(195)
IDBA-UD	IDBA-UD is a *denovo* assembler of metagenomic sequences with uneven depth.	http://i.cs.hku.hk/~alse/hkubrg/projects/idba_ud/	(196)
MetaVelvet	MetaVelvet is a *denovo* assembler of metagenomic short reads.	http://metavelvet.dna.bio.keio.ac.jp/	(197)
RayMeta	RayMeta, a *denovo* assembler of metagenomic reads and taxonomy profiler by Ray Communities.	http://denovoassembler.sourceforge.net/	(198)
MetaGeneMark	MetaGeneMark is a gene coding sequences predictor from metagenomic sequences by heuristic model.	http://exon.gatech.edu/index.html	(199)
GlimmerMG	GlimmerMG is a gene coding sequences predictor from metagenomic sequences by unsupervised clustering.	http://www.cbcb.umd.edu/software/glimmer-mg/	(200)
FragGeneScan	FragGeneScan is a gene coding sequences predictor from short reads.	http://sourceforge.net/projects/fraggenescan/	(201)
CD-HIT	CD-HIT is a tool used for clustering and comparing of sequences of nucleotides or protein.	http://weizhongli-lab.org/cd-hit/	(202)
HMMER3	HMMER3 is a free and commonly used software package for sequence analysis. It is a Hidden Markov based model used to perform sequences alignments. Used for the identification of the homologus nucleotide and protein sequences	http://hmmer.janelia.org/	(203)
BLASTX	Basic local alignment of translated sequences	http://blast.ncbi.nlm.nih.gov/blast/Blast.cgi	(203)
MetaORFA	MetaORFA is applied for the assembly of peptides obtained from predicted ORFs.	Website not available	(204)
MinPath	MinPath is a tool used for reconstruction of pathways from protein family predictions.	http://omics.informatics.indiana.edu/MinPath/	(205)
MetaPath	MetaPath is used for the identification of metabolic pathways that are differentially abundant within the metagenomic samples.	http://metapath.cbcb.umd.edu/	(206)
GhostKOALA	GhostKOALA is KEGG's internal annotator of metagenomes by k-number assignment by GHOSTX searches against a non-redundant database of KEGG genes.	http://www.kegg.jp/ghostkoala/	(207)
RAMMCAP	RAMMCAP is used for the metagenomic functional annotation and data clustering.	http://weizhong-lab.ucsd.edu/rammcap/cgi-bin/rammcap_2d.cgi	(208)
ProViDE	ProViDE is a tool for the analysis of viral diversity in metagenomic samples.	https://omictools.com/provide-tool	(209)
Phyloseq	Phyloseq is a tool-kit for raw reads pre-processing, diversity analysis and graphics production. It is an R, Bioconductor package.	https://joey711.github.io/phyloseq/	(210)
Metagenome Seq	MetagenomeSeq is designed to determine the analysis of differential abundance of 16S rRNA gene in metaprofiling data. It is also designed to address the effects of both under-sampling and normalization of microbial communities on the basis of disease association detection.	http://bioconductor.org/packages/release/bioc/html/metagenomeSeq.html	(211)
Shotgun Functionalize R	Shotgun Functionalize is an R-Package for the functional assessment of metagenomic data. The package includes tools designed for importing, annotating and visualizing metagenomic data generated via high-throughput sequencing.	http://shotgun.math.chalmers.se/	(212)
Galaxy portal	Galaxy portal is a web repository of computational tools that can be run without informatics expertise. It is a graphical interface and free service.	https://usegalaxy.org/	(213)
MG-RAST	MG-RAST an open source web application is used for the automatic phylogenetic and functional analysis of metagenomes. MG-RAST is one of the biggest repositories for metagenomic data. It is a Graphical interface, web portal and free service.	http://metagenomics.anl.gov/	(214)
IMG/M	IMG (Integrated Microbial Genomes) system serves as a community resource for the analysis, functional annotation and phylogenetic distribution of genes and comparative metagenomics. It is a graphical interface, web portal and free in service.	https://img.jgi.doe.gov/	(215)
Phinch	Phinch is an open source, interactive exploratory data visualizing tool intended to alleviate the analysis of meta-omic datasets. The main features of this software are streamlined visualization workflow, sleek user interface, novel exploration of larger datasets. Accessible via web browser.	http://phinch.org	(215)
CAMERA	CAMERA is an important tool that aims to bridge the gaps and to develop methods so as to monitor microbial communities of the oceans. CAMERA's databases incorporate both the genomic and metagenomic datasets, metadata, results from the pre computed analysis and softwares that endorse commanding cross-analysis of the environmental metagenomes.	https://omictools.com/camera-2-tool	(216)
Meta Comp	Meta Comp is a graphical inclusive analysis tool that encompasses a series of statistical analysis approaches along with visualized results for comparative analysis of metagenomics as well as other meta-omics data sets. The software has the features to read files generated via different upstream analysis programs. It has also got the features to automatically choose two-group sample test.	http://cqb.pku.edu.cn/ZhuLab/MetaComp/	(216)

**Table 3:** Comparative workflow of the four most commonly used bioinformatics pipeline for analyzing metagenomic datasets.

	**EBI**	**MGRAST**	**QIIME/QIIME 2**	**MOTHUR**
License	Free open-source	Free open-source	Free open-source	Free open-source
Implementation (release candidate)	Python	Python	Python	C^++^
Current Version available (March 2018)	4.1	4.0.3	1.9.1 and 2017.6.0, respectively	1.39.5
Website	http://www.ebi.ac.uk/metagenomics	http://metagenomics.anl.gov/	http://qiime.org/ and https://qiime2.org/	http://www.mothur.org/
Primary Usage	GUI	GUI	CL and GUI, respectively	CL
Amplicon data Analysis	Yes	Yes	Yes	Yes
Whole genome shotgun analysis	Yes	Yes	Yes but only experimental	No
Sequencing technology compatibility	Sanger, PacBio, Ion Torrent, Illumina, Nanopore	Sanger, PacBio, Ion Torrent, Illumina, Nanopore	Sanger, PacBio, Ion Torrent, Illumina, Nanopore	Sanger, PacBio, Ion Torrent, Illumina, Nanopore
Quality control	Yes	Yes	Yes	Yes
16S rRNA gene Databases searched	Silva, Rfam, MAPSeq, Pfam, TIGRFAM, Prints, Prosite patterns, Gene 3d	Silva, M5RNA, RDP and Greengenes	Greengenes, RDP, Siva and Unite	RDP, Greengenes Silva and Unite
Alignment method	PyNAST, MUSCLE, INFERNAL	BLAT	PyNAST, MUSCLE, INFERNAL	Needleman-Wunsch, Blastn, Gotoh
Taxonomic assignment	UCLUST, BLAST, Mothur, RDP	BLAT	UCLUST, BLAST, Mothur, RDP	Wang/RDP approach
Clustering algorithm	UCLUST, BLAST Mothur, CD-HIT	UCLUST	UCLUST, BLAST Mothur, CD-HIT	Mothur, CD-HIT and adapts DOTUR
Diversity analysis	Alpha and beta	Alpha	Alpha and beta	Alpha and beta
Phylogenetic Tree	YES	YES	FastTree	Clear cut algorithm
Visualization	T, BC, PC, HM, SC, PCA, Krona and Circos	T, BC, PC, HM, SC, PCA, Krona and Circos	T, BC, PC, HM, SC, PCA	T, BC, PC, HM, SC, PCA, Dendrograms, Venn diagrams
Submitted projects as on March 2018	Total: 1,653 Public: 1,503 Private:151	Total: 3,24,846 Public:52,615 Private:272,231	NA	NA

*BC, Bar-Charts; BLAT, Blast like Alignment Tool; CL, Command Line; EBI, European Bioinformatics Institute; GUI, Graphical User Interface; HM, Heat Map; MGRAST, Metagenomic Rapid Annotations using Subsystems Technology; OUT, Operational Taxonomic Unit; PC, Pie-Charts; PCA, Principal Component Analysis; QIIME, Quantitative Insights into Microbial Ecology; RDP, Ribosomal Database Project; SC, Stacked Columns; T, Tabulation*.

## Metagenomic Data Analysis Software: Command Based Vs. Graphical User Interface

**Figure 6:**
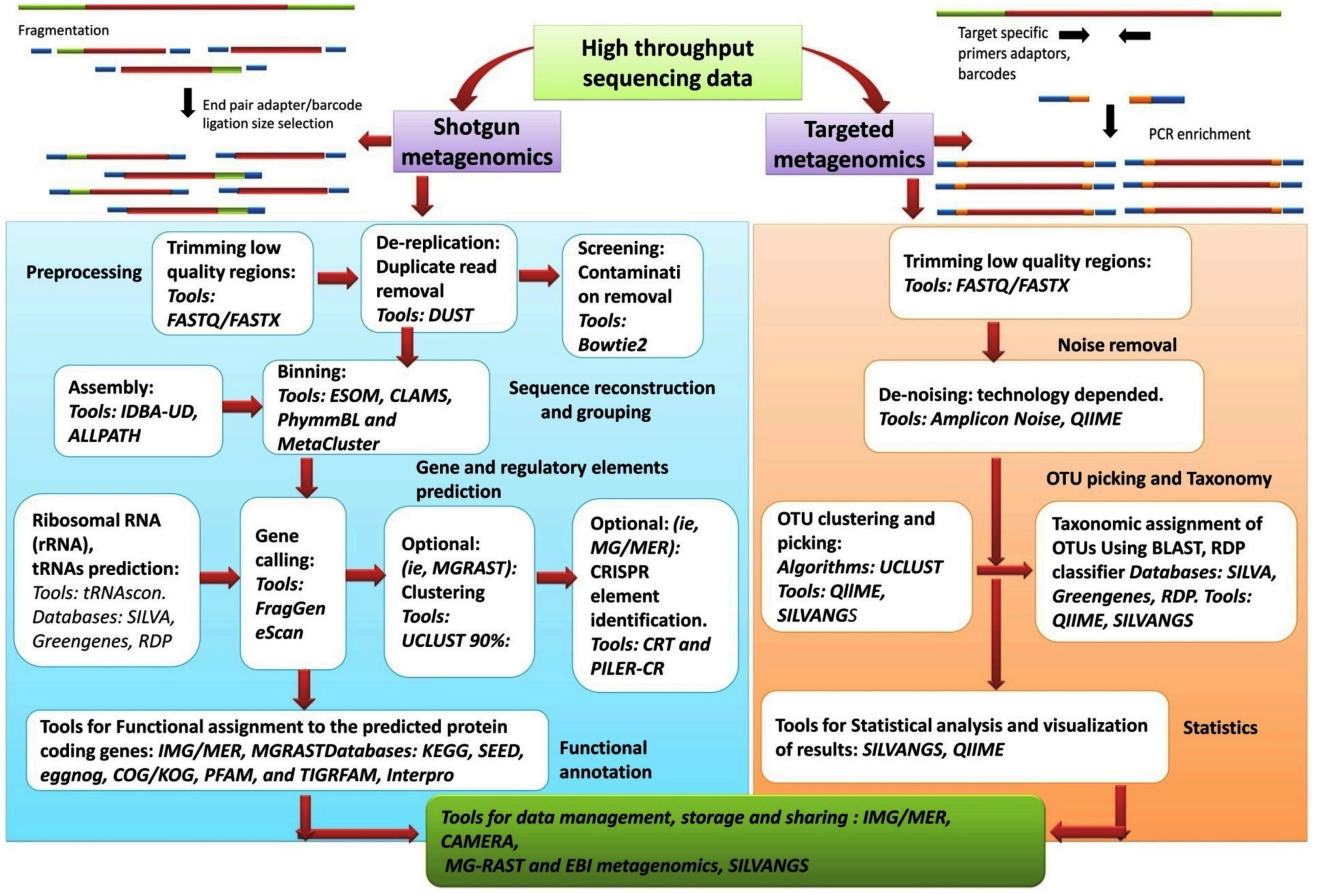
Flow chart of basic metagenomics steps and tools currently in use.

As comprehensive metagenomic studies are becoming more common, they are yielding novel and important insights into the microbial communities in diverse environments; from terrestrial to aquatic ecosystems and from human skin to the human gastrointestinal tract. Advances in NGS have made it more possible than ever for researchers to conduct whole genome sequencing. The analysis of the datasets obtained from NGS is complex and require an intelligent and systematic approach to process the data efficiently. The results obtained from any metagenomic study relies on *in silico* computational tools that can analyse large data sets and can mine and highlight various aspects about the community being examined. Although the tools and databases developed to investigate the taxonomic composition of a microbial community and provide information on the functional aspects of the community are becoming more elaborate and complex, though CLC microbial genomic package offered by Qiagen are good for these analysis. Nanopore sequencing technology has presented an option for an analysis pipeline, with novel options for assembly and annotation. Figure 6, presents the workflow involved in metagenomic analysis, and indicates all the steps and tools used for analyzing the data generated from metagenomic sequencing. The metagenomic pipeline can utilize any of the presented approaches, based on type of sequencing data (targeted metagenomics or shotgun metagenomics). The flowchart summarizes the basic steps that are followed in the analysis pipeline starting from preprocessing of the sequencing data to the final extraction, storage, and presentation of the data. The most popular tools, along with the databases and algorithms employed for the analysis, are indicated.

## Technology and the Changing Landscape of Metagenomic Research

**Figure 7:**
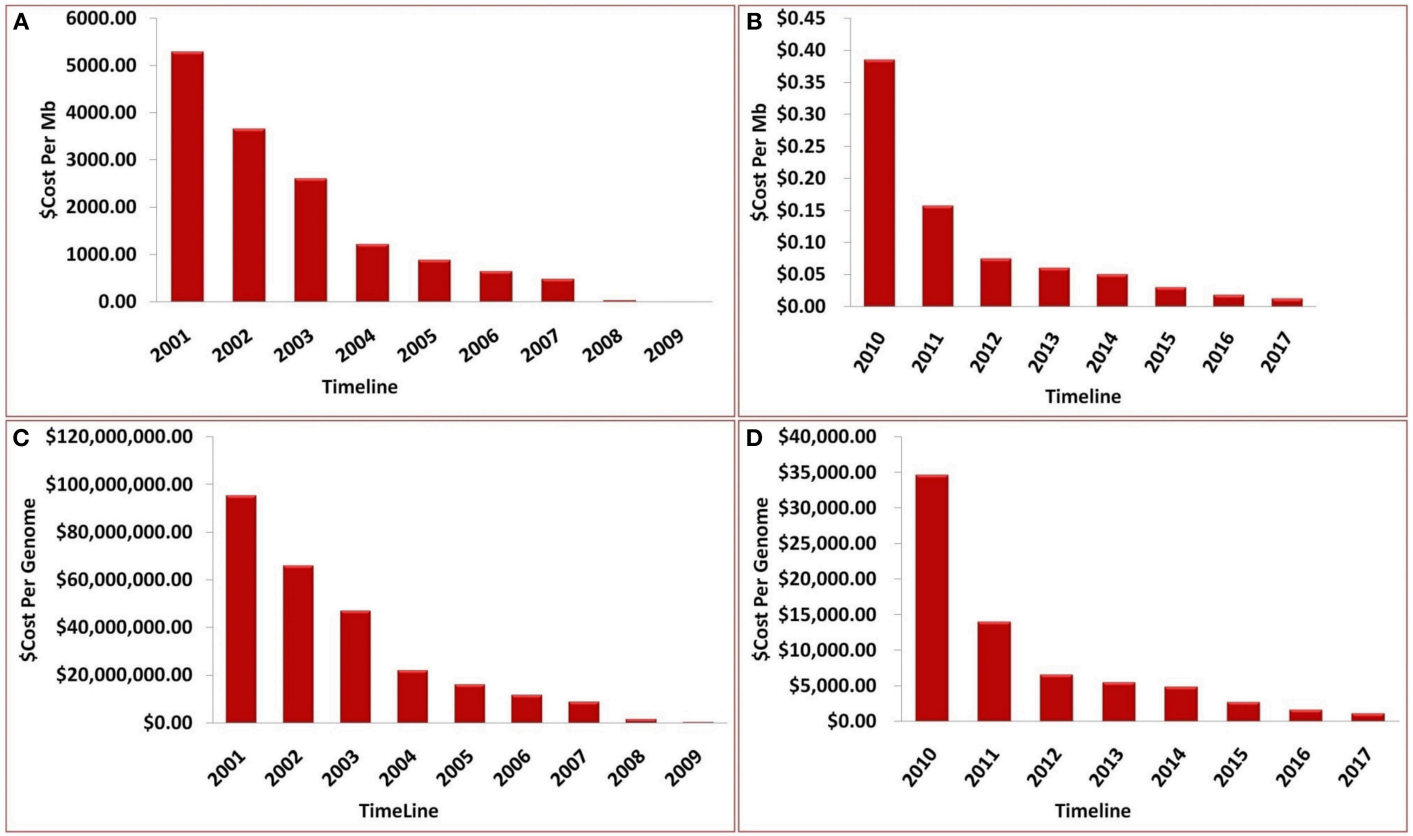
Timeline showing the sequencing cost **(A)** per Mb until year 2009, **(B)** per Mb between year 2009 and 2017, **(C)** per genome until year 2009, **(D)** per genome between year 2009 and 2017.

Over the past decade, advancements in NGS have led to a significant reduction in the cost of genome sequencing. These technological advances have enabled the sequencing of several genomes in a day at a cost of approximately $1,000 per genome (Figure 7). The cost estimates presented in Figure 7 represent (A) cost in U.S, dollars per Mb of sequence data from 2001 to 2009, (B) cost in U.S, dollars per Mb from 2009 to 2017, (C) cost in U.S, dollars per Genome from 2001 to 2009, and (D) cost in U.S, dollars per Genome from 2009 to 2017. Although sequencing is now relatively easy and straight forward, NGS technology is not perfect and errors in the data do occur. Moreover, some regions of the DNA have not been successfully sequenced. The underlying costs associated with different approaches to sequencing genomes are of great importance because they impact the scope and scale of genomic projects. Decreases in sequencing costs have led to the establishment of large collaborative projects with broad goals and individual laboratories targeting more specific questions.

The decreasing cost structure of DNA sequencing has had and will continue to have an impact on genomics and bio-computing. With the size of databases expanding continuously, the translation of data into biological insight is becoming more and more important. As a result, data analysis a more prominent aspect in obtaining information and value from the data (217). Significant analytical efforts are needed to gain useful insights from the generated data. The fields of microbiology, biotechnology, and medicine are already benefiting from genome sequencing efforts, and as costs continue to decrease, the practice of genome sequencing is expected to become almost routine. For example, the Sanger Institute is sequencing the genomes of patients suffering from cancer and rare diseases as part of the 100,000 Genomes Project organized by Genomics England.

Some patients have already benefitted from metagenomic-based diagnoses and treatments, and researchers are continuing to gain more knowledge about the genetic variations that cause a variety of diseases. Sequencing, however, is not the only option for genetic analysis. An important part of the Precision Medicine Initiative, organized by the US National Institute of Health, is to develop a more predictable and possibly less technically complex method of genetic analysis. Sequencing, however, appears to be the only way to comprehensively explore the complex features of DNA that guide the initiation and progression of a number of diseases. Additionally, comprehensive sequencing also helps determine how our DNA keeps us healthy (218).

## Future Perspectives of Metagenomics and Human Health

Though the field of metagenomics pre-dates NGS, modern high-throughput sequencing technologies have greatly transformed this promising field by enabling a comprehensive characterization of all microorganisms present in a sample. As metagenomic approaches become more developed and clinically corroborated, it is expected that metagenomics will be at the forefront as a method for diagnosing infectious diseases. When a complex or unknown infectious disease is encountered, the use of multiple conventional diagnostic tests can potentially lead to unnecessary expenses; more importantly, this can also result in the delay of a diagnosis. Metagenomics can be used to identify potential pathogens, both known and novel, and can also be used to assess the state of an individual's microbiome. As sequencing become easier, faster, and more cost-effective, it will be possible to serially characterize the human microbiota to explore changes that occur in the human microbiome over time. This knowledge could lead to the development of novel medicines and approaches for treating infectious diseases. Indeed, metagenomic studies may become so standard that DNA sequencers could be used in homes to monitor changes in the stool microbiome of an individual to guide the maintenance of health.

## Conclusions

All forms of life on this planet are dependent on microbes. They define an environment and are in turn defined by it. Our understanding of host-pathogen systems, however, is only in its infancy. Over the past two decades, sequencing technology, along with bioinformatic tools, have improved significantly; making it feasible to explore microbial communities residing within diverse hosts. There is a strong recognition that the microbial diversity existing in extreme habitats has largely been unexplored. To gain insight into this “latent” microbial flora, novel methodologies are required. NGS technologies have provided a rapid, cost-efficient means of generating sequencing data and provided sequencing platforms that can be used in large genome-sequencing centers, as well as individual laboratories. Illumina, PacBio, and Applied Biosystems, have all announced upgraded versions of their respective DNA-sequencing platforms. These upgrades will increase high-throughput ability and read length, while at the same time significantly reduce the cost of sequencing per base. These developments will significantly contribute to and provide exciting new opportunities to microbiologists. The integration of several approaches to biological studies will be necessary to answer questions about the diversity and ecology of microbial flora. It is the opinion of the authors of the present review that the development of better bioinformatic tools for analysing metagenomic data is urgently needed. The vast amounts of metagenomic data that will be forthcoming will bring new challenges for analysing, storing, and transferring data. Genome-sequencing centers and laboratories are going to become more dependent on information technology and bioinformatics. Bioinformatic expertise will increasingly be necessary to analyse large amounts of data and to mine the data for useful information about microbial diversity. Metagenomics will play an increasing role in the fields of medicine, biotechnology, and environmental science. The authors hope that this review provides a clear overview of the sequencing platforms and bioinformatic analysis of software that are available, including their high value and limitations.

## Author Contributions

MM prepared the draft of the manuscript under the guidance of AK and SY. AD prepared the illustrations. EA and AH edited the manuscript.

### Conflict of Interest Statement

The authors declare that the research was conducted in the absence of any commercial or financial relationships that could be construed as a potential conflict of interest.

## Abbreviations

PCR: Polymerase chain reaction
NGS: Next generation sequencing
GIT: Gastrointestinal tract
IBD: Inflammatory bowel disease
IBS: Irritable bowel syndrome
SCFA: Short-chain fatty acids
PYY: Peptide YY
HIV: Human immunodeficiency virus
KEGG: Kyoto Encyclopaedia of genes and genomes
GOLD: Genomes Online Database
SBS: Sequencing by synthesis
dNTP: Deoxyribonucleotide triphosphate
PGM: Personal genome machine
ISFET, Ion-sensitive: field-effect transistor
SMRT, Single-molecule: real-time sequencing
ZMW: Zero mode wave
ONT: Oxford Nanopore Technologies
MG-RAST: Metagenomics Rapid Annotation using Subsystem Technology
EBI: European Bioinformatics Institute
QIIME: Quantitative Insights Into Microbial Ecology.
